# Acoustic correlates of perceived personality from Korean utterances in a formal communicative setting

**DOI:** 10.1371/journal.pone.0293222

**Published:** 2023-10-31

**Authors:** Jieun Song, Minjeong Kim, Jaehan Park

**Affiliations:** 1 School of Digital Humanities and Computational Social Sciences, Korea Advanced Institute of Science and Technology, Daejeon, South Korea; 2 Graduate School of Culture Technology, Korea Advanced Institute of Science and Technology, Daejeon, South Korea; 3 KT Corporation, Seongnam-City, South Korea; 4 School of Computer Science, University of Seoul, Seoul, South Korea; McMaster University, CANADA

## Abstract

The aim of the present study was to find acoustic correlates of perceived personality from the speech produced in a formal communicative setting–that of Korean customer service employees in particular. This work extended previous research on voice personality impressions to a different sociocultural and linguistic context in which speakers are expected to speak politely in a formal register. To use naturally produced speech rather than read speech, we devised a new method that successfully elicited spontaneous speech from speakers who were role-playing as customer service employees, while controlling for the words and sentence structures they used. We then examined a wide range of acoustic properties in the utterances, including voice quality and global acoustic and segmental properties using Principal Component Analysis. Subjects of the personality rating task listened to the utterances and rated perceived personality in terms of the Big-Five personality traits. While replicating some previous findings, we discovered several acoustic variables that exclusively accounted for the personality judgments of female speakers; a more modal voice quality increased perceived conscientiousness and neuroticism, and less dispersed formants reflecting a larger body size increased the perceived levels of extraversion and openness. These biases in personality perception likely reflect gender and occupation-related stereotypes that exist in South Korea. Our findings can also serve as a basis for developing and evaluating synthetic speech for Voice Assistant applications in future studies.

## Introduction

As a speaker of any language, we can convey a linguistic message (e.g., words and sentences) to others by articulating speech sounds using the vocal apparatus. However, the speech we produce also provides a significant amount of information about the speaker, including their age, gender, socioeconomic status, and personality [1, 2]. A speaker’s personality can be quickly perceived even from a brief utterance (e.g., “hello”) [3, 4], during which a variety of acoustic cues become available to the listener, including the speaker’s voice quality (e.g., rough voice), the way they talk (e.g., casually or clearly), and their accent (e.g., foreign or regional). Understanding how people judge the personality of an unacquainted person based on these sources of information is particularly important in current society because they frequently communicate with unfamiliar speakers without meeting with them in person (e.g., telephone customer service, phone interviews). Furthermore, this implicit and automatic process of forming a personality impression can have significant impact on our behaviour such as voting decisions or mate choices [5–7].

One of the most common social interactions during which such instantaneous personality impressions are formed occurs when individuals talk to an unfamiliar person at businesses or organizations (e.g., companies, restaurants, hospitals or government offices). This particular communicative situation was the interest of the present study. It is different from other contexts involving unfamiliar speakers because specific speaking styles are typically anticipated from the interlocutors, especially the employees; companies often provide business etiquette training to their employees in which employees learn how to speak to customers using polite language (e.g., use of honorifics, greeting with a higher pitch, answering phone calls politely), and these verbal communication skills affect customer satisfaction [8, 9]. Employees may adopt a certain speaking style when talking to customers in order to convey positive attributes such as being friendly, sincere or confident. Indeed, hiring employees with personality traits that help achieve sales success (e.g., extraversion and conscientiousness [10]) is considered to be an important business tactic [11]. It is interesting to note that this particular communicative situation occurs more widely nowadays, involving both human-to-human and human-to-computer interactions (e.g., AI voice agents).

The present study examined how personality impressions are formed specifically when Korean speakers interact with each other in this context. Korean has an intricate system of honorification, making it crucial for speakers to utilize appropriate honorific forms (e.g., pronouns, verb endings) to convey socially appropriate regard towards the listener [12]. During interactions among unfamiliar individuals in formal communicative settings as described above, it is expected that speakers, especially customer support employees, use appropriate honorific forms to express politeness (e.g.,–*nim* to express that the addressee is their superior). In addition to using an honorific register, speakers can express politeness phonetically. Previous studies have shown that politeness and other related talker attributes are conveyed differently in Korean using acoustic cues. In Winter & Grawunder [13], lower fundamental frequency (f0; acoustic correlate of pitch), a slower articulation rate, and a less breathy voice were found in Korean polite speech. The authors suggested that lower f0 was used for polite speech because it can signal a neutral or sad mood rather than excitement. This result was interesting because an increase in pitch has been reported in other languages for polite speech [14]. Holliday et al. [15] showed that speakers with higher-pitched voices were perceived as being smaller in size, weaker, and more emotional by both Korean and English speakers, supporting [16], but they were perceived as being less and more polite by Korean and English speakers, respectively. These findings suggest that physical size and emotion are related differently to politeness in the two languages, thereby causing the cross-linguistic differences. The same study also found that higher- and lower-pitched speech was perceived as being more friendly and more confident, respectively, in English, whereas Korean showed no such relationship [15]. These previous studies suggest that the judgment of attributes or personality traits based on acoustic and phonetic cues is complex and culturally bounded.

The goal of this study was to explore acoustic correlates of the perceived personality of Korean speakers in formal communicative settings by focusing on utterances of customer service clerks. The current study evaluated perceived personality of the utterances using the Big-Five Model [15]. It is a personality model which has been widely used in many face and voice perception studies [17–19], and assesses personality using 5 dimensions ‐ extraversion, agreeableness, conscientiousness, neuroticism, and openness. Some of the traits have been found to be more easily perceived in voices than others, with extraversion often being the easiest to predict using acoustic cues [20, 21]. Previous research has shown that higher scores of perceived extraversion were associated with higher f0 and higher intensity of the speaker’s voice (i.e., higher-pitched and louder voices) and greater variations in these measures, as well as faster speaking rate [20–23]. The perception of neuroticism has also been shown to be linked with f0 and intensity, with higher values indicating increasing levels of neuroticism [20, 21, 23]. In contrast, utterances with greater pitch dynamics were perceived as being more agreeable [20, 24]. Great pitch dynamics include rises and falls in pitch, showing intonation patterns of an utterance (e.g., pitch accent in English). Intonation is important in language as it conveys the speaker’s emotions (e.g., excitement, comfort, anger), as well as linguistic information (e.g., questions vs. statements) [25]. In addition, speakers with a medium speaking rate were rated as being more benevolent than those with faster or slower speaking rates [26]. Speaking rate has also been found to affect the perception of conscientiousness; speakers who talk faster were perceived to be more conscientious [23, 26]. These prior investigations have been conducted in English, with the exception of Polzehl et al. [20], which examined personality perceptions in German utterances.

The perception of other related personality traits has also been widely investigated in previous research in relation to similar acoustic correlates. Klofstad et al. [6] showed that low-pitched English utterances produced by both male and female speakers were perceived as being more competent and were preferred by voters. A greater f0 variation was also rated as being more competent [24]. More recently, McAleer et al. [3] demonstrated that personality ratings for a brief but socially relevant utterance “hello” can be summarized in a two-dimensional “social space” of “valence”, which is related to likability and trustworthiness, and “dominance”. The acoustic correlates of these personality traits differed for female and male voices. For example, intonation patterns with a greater pitch range and a high average pitch were associated with increasing valence in female and male voices, respectively.

Voice quality, which is associated with the manner of vocal fold variation, also provides important information about the speaker’s identity and emotional states, as well as being used for making phonemic contrasts (e.g., the same vowel with breathy, creaky and modal phonations is used to differentiate meaning in Jalapa Mazatec; [27]). Compared to modal voice, breathy voice is produced with the vocal folds more spread, and creaky voice with the vocal folds more constricted. Both breathy and creaky voices contain more noise than modal voice because of the aspiration noise and irregular pitch they contain, respectively [28].

The effect of voice quality on voice attractiveness has been relatively well-studied. For example, studies have found that breathiness in women’s voices is perceived as attractive. In addition, low perceptual hoarseness is perceived as attractive, whereas creaky voice is generally perceived as unattractive (see [29] for a review). However, our understanding of how a speaker’s voice quality affects the perception of their personality is relatively limited. Waaramaa et al. [30] investigated the relationship between voice quality and perceived personality; they obtained speech produced with varying voice qualities by asking speakers to manipulate the manner of phonation (e.g., normal, breathy, tense, creaky, nasalized). Their results found that creaky voice was associated with introversion, unattractiveness, and a lack of enthusiasm. Breathy voice was rated by native speakers (Finnish) as indicating negative personality traits such as being unpleasant, unbalanced, and untrustworthy though the opposite was true for non-native listeners. Vinciarelli et al. [23] examined spectral measures related to voice quality (e.g., spectral centroid) in relation to personality perception, which found that brighter voice was perceived as being less agreeable. In McAleer et al. [3], decreasing Harmonics-to-Noise Ratios (HNRs) were associated with higher valence scores for both male and female speakers; lower HNRs reflect the roughness of the voice (i.e., greater aperiodicity in the harmonics). The authors suggested that it was because older voices were perceived as being more friendly or trustworthy. On the whole, it will be worthwhile to investigate the effect of voice quality further, independent of age effects, by examining inter-speaker variability that arises in natural utterances.

The present study investigated how acoustic characteristics of speech, including prosodic, segmental, and vocal characteristics, affect personality judgements of speakers who talk in customer service scenarios. To this end, we used spontaneous speech extracted from conversations that took place between a customer and an employee in an experimental setting. Speakers may adopt a specific speaking style as a customer service employee to convey more positive traits that are appropriate for the social context (e.g., professional or kind), which needs to be examined not only using global, prosodic properties of speech (e.g., f0), but also using segmental (e.g., vowels) and voice quality features. It is possible that customer service employees increase speech production effort when speaking to customers; some of the phonetic characteristics of polite speech are similar to those of clear speech, a speech register that is adopted to facilitate the listener’s speech comprehension in adverse conditions (e.g., in noisy environments; for a review, see [31]). Specifically, Korean speakers slowed down their speaking rate in a formal speech register [13], and they were also shown to hyperarticulate vowels in polite speech (i.e., when speaking to a professor compared to speaking to a friend; [32]). However, the link between personality judgements and vowel hyperarticulation has not been previously investigated. The current study examined vowel production and articulation rate as well as other prosodic characteristics (e.g., average f0, f0 range) to understand how these speech characteristics are associated with the different personality traits (e.g., agreeableness, conscientiousness).

Moreover, we aimed to conduct a careful investigation of voice quality to understand how it affects the perception of personality, which is poorly understood in previous research. In the present study, differences in voice quality were measured more accurately in terms of a series of acoustic parameters extracted from the psychoacoustic model of voice quality [33]; the different variables need to be examined together to correctly understand the voice quality [28]. Moreover, it is necessary to investigate the effect of voice quality across different speakers as well as within [30] because voice quality provides an important source of information about speaker identity [34]. Throughout this study, we also investigated the relationship between these acoustic parameters and perceived personality separately for male and female speakers because there is ample evidence from previous research suggesting that acoustic correlates of personality or attractiveness differ between male and female speakers [3, 29].

Understanding speech characteristics of employees during formal interactions with customers, and identifying the acoustic parameters that convey positive personality traits will provide valuable information when developing speech synthesis systems for a Voice Assistant (VA), an artificial intelligence system that communicates with users using spoken language (e.g., Amazon’s Alexa, Apple’s Siri). Nowadays, some applications offer various voice options to choose from (e.g., KT AI Voice Studio in South Korea), and services that allow users to build custom voices are also becoming popular [35]. In line with the increasing diversity in synthetic voices, understanding the acoustic markers of positive personality traits in the speech of customer service staff will contribute to the development of synthetic speech that projects a more positive image (e.g., competence) to users in VA applications. Similarly, our work is also relevant to speech-based automatic personality recognition, which can be used for many computer applications such as speech dialogue systems [36, 37].

Taken together, the current study extends previous voice personality research to a social context in which specific personality traits or speaking styles are desirable (e.g., politeness, competence). We investigated this using spontaneous utterances produced in Korean. Using spontaneous speech samples is normally difficult as they contain a lot of variabilities that cannot be controlled for (e.g., words and sentence structures) because speakers naturally converse with each other rather than reading a script. In contrast, they have different segmental and prosodic characteristics from read speech [38], and one can find some speech characteristics more easily in spontaneous speech, such as connected speech processes, phonetic reduction [39], and speech style adaptations depending on the needs of the situation (e.g., hyperarticulation). We may be able to find acoustic variability (e.g., variability in f0) that would not be found in read speech [40]. We thus devised a new method for eliciting spontaneous speech from subjects while they played the role of a customer service employee talking to a customer, and this method controlled for the lexical items and syntactic structures of their utterances. Using this new method, the present study aimed to explore a more comprehensive set of acoustic cues–prosodic, segmental, and vocal characteristics that could reflect the speaker’s personality. We performed a personality perception experiment in which subjects rated perceived personality of the utterances in terms of the 5 personality traits (extraversion, agreeableness, conscientiousness, neuroticism, and openness), and we investigated the relationship between the acoustic parameters and the personality ratings.

## Methods

### Speech elicitation task

This study was approved by the IRB committee of the Korea Advanced Institute of Science and Technology (KH2022-013), and all participating subjects provided written informed consent. Thirty native speakers of Korean (15 female, 15 male) with mean age of 25 (range: 21 ∼ 37) took part in a speech elicitation task in a sound-proof booth. They reported growing up in Seoul or Gyeonggi, indicating that they all spoke Seoul Korean. During the task, the subjects played the role of a customer service employee assisting a customer while the researcher (the second author) sitting outside of the booth was playing the role of the customer. They were talking to each other over the phone in order to create a realistic phone conversation situation. The researcher made customer inquiries regarding the business (e.g., reservations, locations, deliveries). The subjects were given answers to those questions on the screen which were written in simplified language (i.e., noun phrases) as shown in Table 1, and they responded to the inquires naturally based on that information. The subjects’ utterances were recorded throughout the task with a RODE NT1 condenser microphone (sample rate: 44100Hz, quantization rate: 16 bit).

**Table 1:** Examples of information given to subjects during the speech elicitation task by situation type.

Situation	Response																			
Location guide	cihachel	1hosen			Kyotayyek,				6pen						chwulkwu				aph,	
	지하철	1호선			교대역,				6번						출구				앞,	
	subway	one line			Gyodae station,				six number						exit				front,	
	Kana pilting			2chung.																
	가나빌딩			2층.																
	Ghana building			two floor.																
	*"Second floor*, *Gana building in front of Exit 6 of Gyodae Station on Subway Line 1*.*"*																			
Parking guide	Kana pilting			ciha			2chung,				3chung						cwuchacang.			
	가나빌딩			지하			2층,				3층						주차장.			
	Ghana building			basement			two floor,				three floor						parking lot.			
	ciha 1chung-un						cwucha		pwulka.											
	지하 1층은						주차		불가.											
	basement one floor-TOP						parking		impossible											
	*“B2 and B3 of Ghana building*. *Impossible to park on B1*.*”*																			
Opening hours guide	yencwungmwuhyu.					yengepsikan:				ocen11:00-ohwu9:00										
	연중무휴.					영업시간:				오전11:00-오후9:00										
	all year round open.					business time:				morning 11:00 ‐ afternoon 9:00										
	*“Open all year round*. *Opening hours*: *11*:*00 a*.*m*. *to 9*:*00 p*.*m*.*”*																			
Opening dates inquiry	2wel		1il	yengep,			yengep sikan:						ocen11:00-ohwu9:00							
	2월		1일	영업,			영업 시간:						오전11:00-오후9:00							
	February		first	business,			business time:						morning 11:00 ‐ afternoon 9:00							
	*“Open on the 1*^*st*^ *of February*. *Opening hours*: *11*:*00 a*.*m*. *to 9*:*00 p*.*m*.*”*																			
Take-away inquiry	phocang		kanung.			sacang-nim-ul						thongha-yse-man							kanung.	
	포장		가능.			사장님을						통해서만							가능.	
	take-away		possible.			owner-HON-OBJ						use-CONJ-only							possible.	
	sacangnim-kwa			cenhwathonghwa					wenha-nyako							mwut-ki.				
	사장님과			전화통화					원하냐고							묻기.				
	owner-COM			phonecall					want-QUO							ask-NOM.				
	*“Take-away possible*. *Only through the (business) owner*. *Ask if they want to talk to the owner on the phone*.*”*																			
Delivery inquiry	paytal	pwulkahata-ko						cental			hwu			sakwaha-ki.						
	배달	불가하다고						전달			후			사과하기.						
	delivery	impossible-QUO						to pass on			after			apologise-NOM.						
	phocang-un				kanung,			phocang				cinhayng						wenha-nyako		mwut-ki.
	포장은				가능,			포장				진행						원하냐고		묻기.
	Take-away-TOP				possible,			take-away				to proceed						want-QUO		ask-NOM.
	*“Tell them that the delivery service is unavailable*, *then apologize*. *Take-away possible*. *Ask if they want a take-away*.*”*																			

The information given to the subjects in Korean (1^st^ row: written with the Roman alphabet using the Yale system, 2^nd^ row: the Korean alphabet, Hangul) is followed by English glosses (3^rd^ row) and translations; TOP-topic, HON-honorific, OBJ-object, CONJ-conjunctive, COM-comitative, QUO- quoted statement/question, NOM-nominal form

Using this method, we were able to elicit spontaneous speech in customer service scenarios while controlling lexical items of the utterances to a large extent. That said, utterances often differed across speakers in verb endings and word orders even when conveying the same linguistic message. We found 49 unique utterances that were identical across multiple speakers. Sentences lacking case particles (e.g., OBJ *-rul*, NOM *-ka*, *-i*) were considered the same as those with the particles, as case particles are commonly omitted in Korean. There were also a few utterances in which synonyms with no difference in meaning were used (e.g., a-hop-si sam-sip-pwun “nine thirty’ vs. a-hop-si pan “half past nine”); they were considered to be identical. The number of speakers who produced each of the 49 utterances differed between 3 and 9. We selected a total of 200 utterances produced by 27 speakers (14 female, 13 male) to be used for the personality rating experiment.

### Personality rating task

Thirty native speakers of Korean (13 male, 17 female), who did not participate in the speech elicitation task, took part in the personality rating task via the online experiment platform Gorilla. They were adults between 19 and 29 years of age (mean: 22.9). We used the Korean version of the Big-Five Inventory (BFI) 10 [41], which contained statements of the BFI-10 translated into Korean (Table 2) [42].

**Table 2:** Big Five Inventory-10 [42].

Personality trait	Statement
Extraversion	…is reserved. …is outgoing, sociable
Agreeableness	… is generally trusting. …tends to find fault with others.
Conscientiousness	…tends to be lazy. …does a thorough job.
Neuroticism	…is relaxed, handles stress well. …gets nervous easily.
Openness	…has few artistic interests. …has an active imagination

The subjects listened to each of the 200 utterances and rated the degree of perceived personality on a 5-point Likert scale (1: strongly disagree, 5: strongly agree). After listening to each utterance, they answered the 10 questions that were presented in a random order. The subjects were allowed to listen to the utterances more than once if they wanted. They were given 3 practice trials to familiarize themselves with the task before starting the experiment. The entire experiment was divided into 8 blocks (25 utterances). The subjects were encouraged to take a break between blocks. The blocks were created so that subjects did not hear the same utterance type more than once within each block. The order of stimuli within each block and the order of blocks were randomized for each subject.

### Acoustic measurements

For acoustic measurements of the utterances, phone boundaries were found automatically using a Korean forced aligner [43] and were then manually corrected. Table 3 shows acoustic variables that were examined in the current study. F0, formants, energy and spectral tilt, and spectral noise variables were all extracted using VoiceSauce [44]. The F0 measurement from the STRAIGHT algorithm [45] was used for this study.

**Table 3:** Acoustic variables examined in this study.

Type		Acoustic variables
Voice	Rate of vocal fold vibration and energy	F0, Energy
	Harmonic source spectral slope	H1*-H2*, H2*–H4*, H4*–H2kHz*, H2kHz*–H5kHz*
	Inharmonic source noise	HNR05, HNR15, HNR25, HNR35, CPP
Others		F0 range
		Articulation rate
		Euclidean distance
		Formant dispersion

We chose the voice parameters based on the psychoacoustic model of voice quality [33]. First, measures of spectral noise were extracted; Harmonics-to-Noise Ratios (HNR) is a ratio between the harmonic and inharmonic components of the signal, which reflects aperiodicity in the harmonics. HNRs can be measured between 0-500Hz, 0-1500Hz, 0-2500Hz, and 0-3500Hz (HNR05, HNR15, HNR25, and HNR35, respectively), with higher values indicating a more modal voice (i.e., less creaky or less breathy, or less hoarse). Breathy and creaky voice can both lower HNR because they are noisier than modal voice (see [28] for a review). Cepstral Peak Prominence (CPP) is highly correlated with HNR, reflecting aperiodicity; it is larger for modal phonations and smaller for breathy phonations (can also be smaller for creaky phonations). That is, the amount of noise in the voice is measured with both HNR (below 3500Hz) and CPP for which higher frequencies are given greater weight [46].

We also extracted four harmonic source spectral tilt measures, H1*-H2*, H2*–H4*, H4*–H2kHz*, H2kHz*–H5kHz*. H1, H2 and H4 refer to the first, second and fourth harmonics, and H2kHz and H5kHz refer to the harmonics closest to 2000 Hz and 5000 Hz, respectively. Asterisks indicate that the values of harmonics were corrected to account for the effect of formants. Spectral tilt measures generally reflect the degree of glottal constriction, with higher values indicating a breathier or less constricted (i.e., modal) voice [28, 33]. Because it is difficult to know whether a higher value indicates a breathier or more modal voice, it is often interpreted together with an HNR. For example, a higher H1-H2 and a higher HNR indicate a more modal than creaky voice, while a higher H1-H2 and a *lower* HNR indicate a breathier than modal voice [28].

Principal Component Analyses (PCA) were conducted to convert these related acoustic variables into a smaller number of significant, uncorrelated components. With PCA, the most variance of the data is accounted for by the first component, and each subsequent component accounts for a lesser variance. This method was particularly necessary for the voice quality measures as they are highly correlated with one another, and a similar approach was used in previous studies of voice quality [40, 47]. Only those parameters related to the vocal folds directly (i.e., rate and manner of vocal fold vibration; the variables belonging to the “voice” type in Table 3) were thus included in PCAs.

Psych package [48] was used in R to carry out PCAs. The 11 voice-related measurements listed in Table 3 were extracted every 5 milliseconds during vowels and approximants and were z-score normalized by gender. PCAs were performed separately for female and male speakers. An oblique rotation was used because these variables were expected to correlate with one another to some extent. After inspecting scree plots, the first 5 principal components were extracted which had an eigenvector greater than 1, following Kaiser’s criterion [49].

To measure articulation rate, average time spent producing each syllable was computed for each utterance with pauses and fillers excluded. As shown in Table 3, we examined two f0-related variables; the f0 values extracted from the utterances were included in PCAs, and f0 range was also computed, which was the difference between the 95th and the 5th percentile of all the f0 values in each utterance. Formant dispersion was also calculated for each utterance, which was the average distance between formants; the average difference between F2 and F1, between F3-F2, and between F4-F3. Formant dispersion is associated with the speaker’s vocal track length and body size [50]. Studies have shown that it is related to voice attractiveness [51]; men prefer female voices with wide formant dispersion because it projects a small body size.

Lastly, the Euclidean distance between /i/ and /a/ was computed in an F1-F2 space for each utterance in order to measure the degree of vowel expansion. Formant frequencies were taken at vowel midpoint. In the current study, vowels were not elicited in specific phonetic environments because the utterances were produced spontaneously. /i/ and /a/ were the most frequently occurring vowels that were found in all 200 stimuli except for 4 which had no occurrences of /i/. We thus calculated the Euclidean distance between the two vowels; there were many utterances without any productions of /u/, so it was not included in the calculation. While /i/ and /a/ occurred frequently in the stimuli, they were produced in different phonetic environments and positions (e.g., phrase initial) in each utterance, but this variation was explained in a mixed-effects model analysis by including utterance as a random effect (see Results).

## Results

### Personality rating task

**Fig 1:**
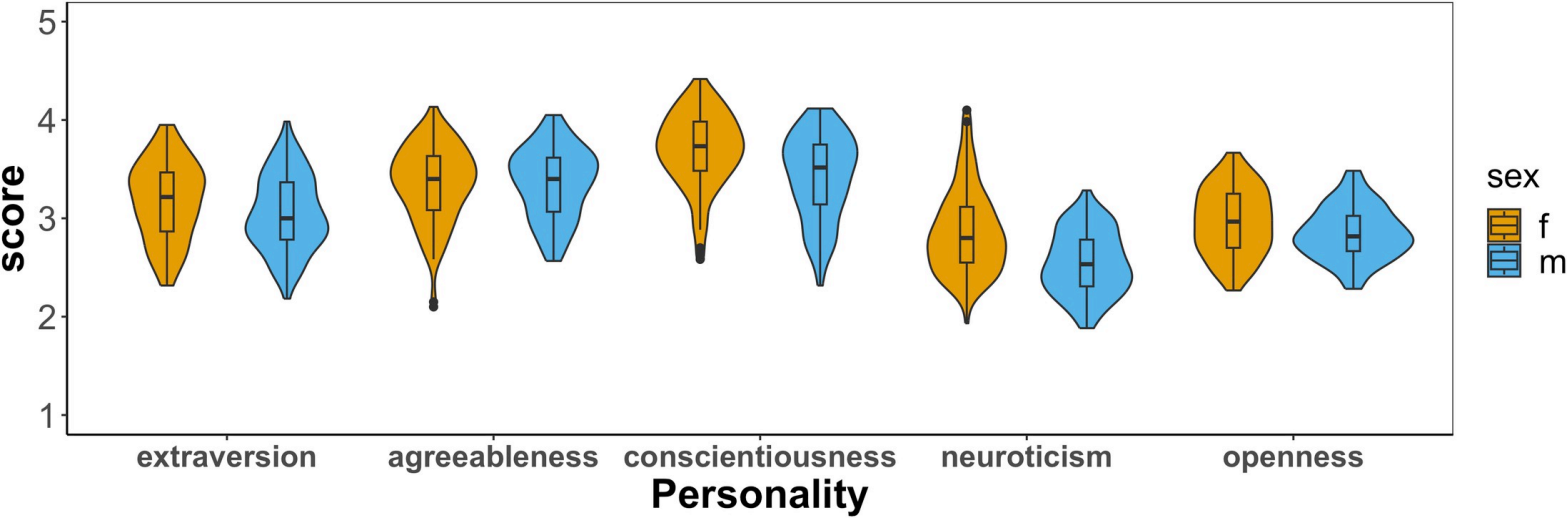
Violin plots of scores for the five personality traits by female and male speakers.

The results of the perception rating task are displayed in Fig 1. The scores of neuroticism were lower than those of other traits. This was likely because the speakers were mostly conveying neutral information in a polite manner thus not showing negative emotions or attitudes related to neuroticism (e.g., anxiety, anger). The scores of conscientiousness and agreeableness were higher than the other traits; those two traits are associated with desired personality traits in customer service employees–being polite, warm, and cooperative while being efficient and capable for the job. A linear mixed-effects model analysis was performed using Lme4 package in R [52] with the score (1∼5) as the dependent variable and with speaker sex and personality trait as fixed effects. Utterances (i.e., 49 unique utterance types), subjects, and speakers were included in the model as crossed random effects. The significance of each factor was obtained by building the model in a stepwise manner (i.e., comparing the model with and without the relevant factor). The results confirmed that the main effect of personality trait was significant, χ^2^(4) = 3736.4, p < 0.001. A post-hoc test using the Multcomp package [53] verified that all pairs of the five personality traits were significantly different, p < 0.001. The interaction of speaker sex and personality trait was also significant, χ^2^(4) = 144.58, p < 0.001. A post-hoc test found that the scores of female speakers were significantly higher than those of male speakers in the conscientiousness (β = 0.250, SE = 0.068, z = 3.689, p < 0.001) and neuroticism ratings (β = 0.289, SE = 0.068, z = 4.263, p < 0.001). The main effect of speaker sex was also significant, χ^2^(1) = 4.59, p = 0.032.

### Principal component analysis

**Fig 2:**
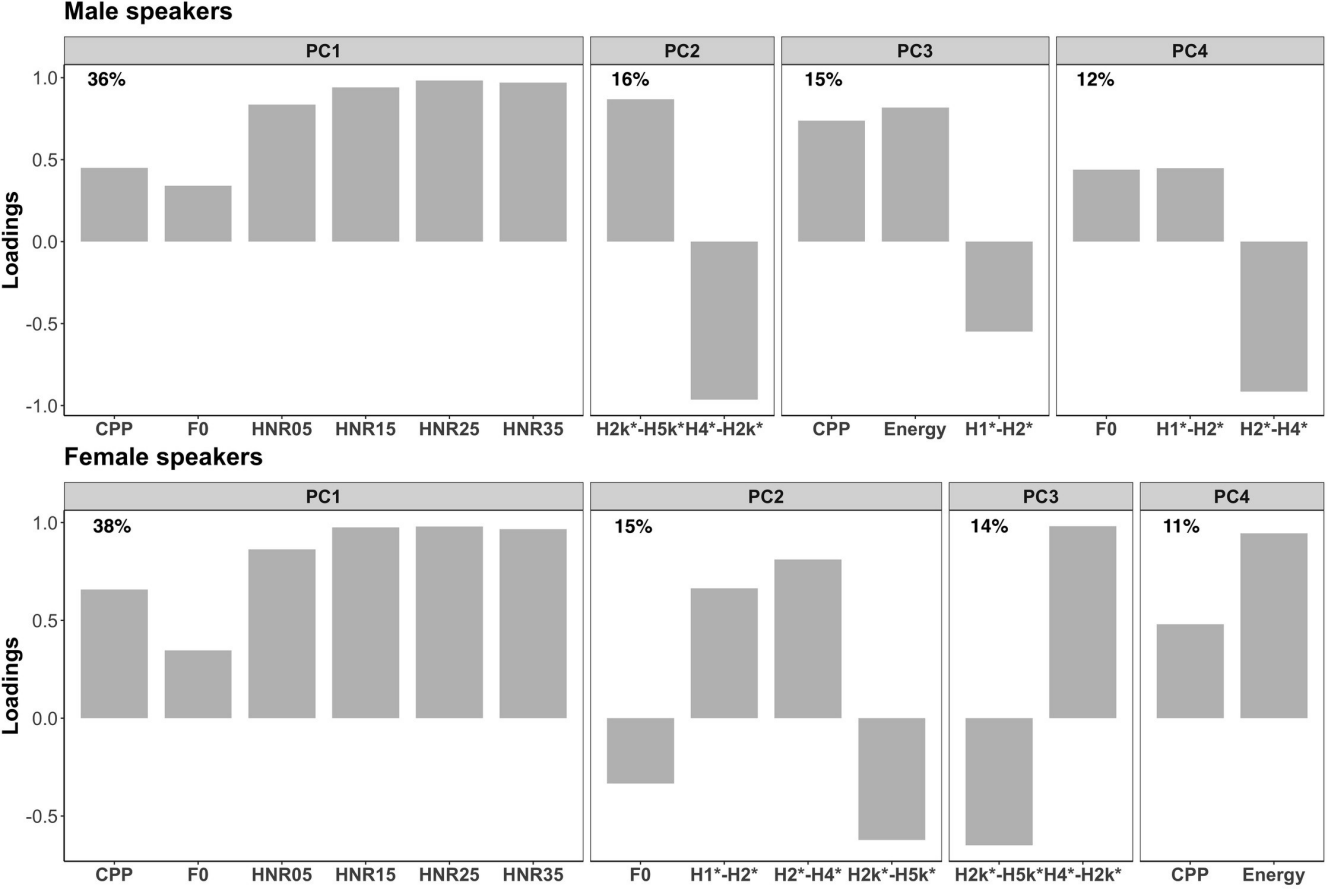
Proportions of the variance explained and loadings of each acoustic variable for each PC by male (top) and female speakers (bottom).

The proportion of the acoustic variance that is explained by each of the extracted components is shown in Fig 2. Only the 11 variables belonging to the “voice” category in Table 3 were included in the PCA analyses, and the four extracted principal components explained 79% and 78% of the acoustic variance of the male and female speakers’ data, respectively. The same figure also displays rotated loadings of each acoustic variable in each component. It displays loadings over 0.32 because only those variables are considered to account for a principal component [54]. After the PCA analyses, a data point along the axes of each acoustic variable (i.e., the original data) appeared as a point along the rotated principal component axes (i.e., PC1, PC2, PC3, PC4); the positions along the new axes are called factor scores, which were used for the main analysis conducted for investigating the relationships between the acoustic variables and the personality ratings.

### Main analysis

Linear mixed-effects model analyses were performed in R for each of the 5 personality ratings, for female and male speakers separately. Factor scores (i.e., rotated data) of the 4 principal components and the 4 additional acoustic variables (i.e., F0 range, articulation rate, formant dispersion, Euclidean distance) were all z-score normalized by gender and were added to each model as fixed effects. Speaker and utterance were included as crossed random effects. The lmerTest package [55] was used to compute the significance of the factors.

For ratings of extraversion for male speakers, f0 range was found to be a significant factor, suggesting that male speakers who spoke with a wider pitch range received higher extraversion scores (Table 4). No other variables were significant in the model of male speakers. F0 range was likewise a significant factor explaining extraversion scores of female speakers (Table 4); female speakers whose pitch range was wider were perceived as being more extroverted. This result is consistent with the finding of previous research [20]. For the ratings of female speakers, PC2 and PC3 were both significant with a negative and a positive slope (b), respectively. As shown in Fig 2, PC2 contains 3 spectral tilt measures (H1*-H2*, H2*–H4*, H2kHz*–H5kHz*) and f0. H1*-H2* and H2*–H4* had a positive loading, and f0 had a negative loading. However, it is difficult to understand why H2kHz*–H5kHz* had a negative loading. Garellek et al. [56] showed that H2kHz*–H5kHz* can be affected by other factors such as high-frequency noise, and listeners’ sensitivity to this acoustic variable is less robust than that of the other spectral tilt variables. Davidson [57] also found that values of H2*–H4* and H2kHz*–H5kHz* for modal and creaky voices did not always go in the expected direction. Although it may be difficult to determine what this component represents in the current study, the result suggested that this voice quality associated with source spectral shape, possibly breathy voice with low pitch, reduces the degree of extraversion perceived from the voice. However, it is also difficult to determine whether higher values of H1*-H2* and H2*–H4* would indicate breathy or modal voice without the noise variables (e.g., HNRs) [28].

**Table 4:** Results of the mixed-effects analysis for the ratings of extraversion.

		Estimate	Std. Error	df	t value	Pr(>|t|)
**Male**	(Intercept)	3.034	0.087	11.788	34.892	0
	PC1	-0.024	0.046	70.888	-0.518	0.606
	PC2	0.061	0.032	75.416	1.873	0.065
	PC3	0.048	0.044	68.761	1.084	0.282
	PC4	0.044	0.034	73.776	1.29	0.201
	**F0range**	0.078	0.032	72.916	2.409	0.019*
	ar	-0.038	0.035	74.639	-1.092	0.278
	ed	0.026	0.031	72.549	0.831	0.409
	fdisp	-0.017	0.042	76.817	-0.411	0.682
**Female**	(Intercept)	3.186	0.085	11.243	37.684	0
	PC1	0.053	0.041	93.831	1.276	0.205
	**PC2**	-0.107	0.049	91.816	-2.178	0.032*
	**PC3**	0.079	0.04	90.178	2.008	0.048*
	PC4	-0.014	0.041	95.75	-0.35	0.727
	**F0range**	0.086	0.033	87.231	2.624	0.01*
	ar	-0.009	0.039	76.513	-0.231	0.818
	ed	-0.002	0.034	86.455	-0.055	0.956
	**fdisp**	-0.11	0.038	91.302	-2.864	0.005**

p-value < 0.001 = ***, p-value < 0.01 = **, p-value < 0.05 = *, p-value > 0.05 = n.s.; ar: articulation rate, ed: Euclidean distance, fdisp: formant dispersion

In contrast, scores of extraversion increased with increasing values of PC3 which contained H2kHz*–H5kHz* with a negative loading (-0.65) and H4*–H2kHz* a positive loading (0.98). The positive correlation between PC3 and H4*–H2kHz* suggests that more modal or breathier voice increases the likelihood of someone’s voice being perceived as being more extroverted. It is difficult to determine whether a higher H4-*H2kHz indicates modal or breathy voice with the spectral tilt measure alone, and why H2kHz*–H5kHz had a negative correlation with PC3 as explained above. In addition, formant dispersion, which is associated with the speaker’s vocal track length and body size [50], was also significant, suggesting that female speakers with larger values of formant dispersion (i.e., shorter vocal tract length and smaller body size) received lower extraversion scores.

None of the acoustic variables were found to be significant for ratings of agreeableness of male speakers (Table 5). In contrast, articulation rate was found to be significant for female speakers with a positive slope (Table 5), indicating that female speakers who spoke more slowly were rated as sounding more agreeable.

**Table 5:** Results of the mixed-effects analysis for the ratings of agreeableness.

		Estimate	Std. Error	df	t value	Pr(>|t|)
**Male**	(Intercept)	3.378	0.066	12.344	51.069	0
	PC1	-0.064	0.052	64.938	-1.235	0.221
	PC2	-0.005	0.039	80.348	-0.125	0.901
	PC3	0.021	0.048	50.145	0.439	0.663
	PC4	-0.049	0.043	78.179	-1.157	0.251
	F0range	0.04	0.038	77.513	1.052	0.296
	ar	-0.007	0.043	81.961	-0.164	0.87
	ed	0.008	0.038	74.064	0.2	0.842
	fdisp	-0.023	0.047	58.317	-0.491	0.625
**Female**	(Intercept)	3.335	0.078	12.376	42.919	0
	PC1	-0.025	0.043	89.312	-0.59	0.557
	PC2	0.022	0.051	88.835	0.429	0.669
	PC3	0.02	0.041	88.977	0.493	0.623
	PC4	-0.028	0.043	94.203	-0.663	0.509
	F0range	0.001	0.034	80.916	0.038	0.97
	**ar**	0.104	0.04	66.211	2.592	0.012*
	ed	-0.027	0.036	84.392	-0.743	0.459
	fdisp	-0.031	0.04	93.215	-0.782	0.436

p-value < 0.001 = ***, p-value < 0.01 = **, p-value < 0.05 = *, p-value > 0.05 = n.s.; ar: articulation rate, ed: Euclidean distance, fdisp: formant dispersion

Articulation rate was a significant predictor of conscientiousness scores of male speakers (Table 6). In contrast to the result that we found with agreeableness scores of female speakers, this result suggested that male speakers who spoke faster received higher conscientiousness scores (a negative slope). This is consistent with the previous research [23, 26]. For female speakers, however, PC1 was a significant factor explaining conscientiousness (Table 6). PC1 of female speakers contained CPP, HNRs, and f0 (Fig 2). This result thus suggested that female speakers whose voice was produced with less noise (i.e., modal rather than creaky or breaky voice) and higher pitch received higher conscientiousness scores. Effects of voice quality have been poorly understood in previous research, but this novel finding demonstrates that more modal voice increases conscientiousness perceived from female voices compared to creaky or breathy voice.

**Table 6:** Results of the mixed-effects analysis for the ratings of conscientiousness.

		Estimate	Std. Error	df	t value	Pr(>|t|)
**Male**	(Intercept)	3.476	0.092	12.562	37.694	0
	PC1	-0.024	0.056	72.991	-0.417	0.678
	PC2	0.075	0.04	77.984	1.865	0.066
	PC3	0.065	0.053	62.994	1.23	0.223
	PC4	-0.015	0.043	76.045	-0.355	0.723
	F0range	0.014	0.04	73.588	0.345	0.731
	**ar**	-0.095	0.044	77.808	-2.179	0.032*
	ed	-0.002	0.039	74.947	-0.05	0.96
	fdisp	-0.036	0.051	75.754	-0.706	0.483
**Female**	(Intercept)	3.688	0.071	12.734	51.92	0
	**PC1**	0.098	0.041	87.296	2.406	0.018*
	PC2	0.078	0.049	88.834	1.605	0.112
	PC3	0.003	0.039	88.799	0.083	0.934
	PC4	0.016	0.041	93.425	0.403	0.688
	F0range	0.051	0.033	80.178	1.57	0.12
	ar	0.045	0.038	67.778	1.18	0.242
	ed	-0.037	0.034	84.942	-1.078	0.284
	fdisp	-0.007	0.039	94.38	-0.179	0.858

p-value < 0.001 = ***, p-value < 0.01 = **, p-value < 0.05 = *, p-value > 0.05 = n.s.; ar: articulation rate, ed: Euclidean distance, fdisp: formant dispersion

For male speakers, none of the acoustic variables were found to be significant for neuroticism scores (Table 7). The effect of articulation rate was close to being significant with a p-value of 0.053. This suggests a tendency that male speakers who spoke faster received higher neuroticism scores. For female speakers, the effect of articulation rate was clearer (Table 7), suggesting that the faster the articulation rate, the higher neuroticism scores they received. PC1 was also significant. This demonstrates that female speakers who talk with modal rather than creaky or breathy voice with high pitch are perceived as being more neurotic.

**Table 7:** Results of the mixed-effects analysis for the ratings of neuroticism.

		Estimate	Std. Error	df	t value	Pr(>|t|)
**Male**	(Intercept)	2.551	0.056	9.119	45.772	0
	PC1	0.051	0.045	55.901	1.14	0.259
	PC2	0.038	0.035	80.382	1.103	0.273
	PC3	0.016	0.042	46.116	0.385	0.702
	PC4	0.047	0.038	75.352	1.242	0.218
	F0range	-0.05	0.034	78.547	-1.472	0.145
	**ar**	-0.074	0.038	80.725	-1.963	0.053
	ed	0.006	0.033	68.924	0.189	0.851
	fdisp	-0.035	0.041	49.542	-0.845	0.402
**Female**	(Intercept)	2.88	0.068	13.374	42.129	0
	**PC1**	0.145	0.042	79.587	3.473	0.001**
	PC2	-0.013	0.052	94.234	-0.246	0.806
	PC3	-0.005	0.042	94.845	-0.124	0.901
	PC4	0.055	0.042	90.771	1.299	0.197
	F0range	0.042	0.035	93.246	1.188	0.238
	**ar**	-0.169	0.042	80.018	-4.033	0***
	ed	0.013	0.037	91.044	0.358	0.721
	fdisp	0.021	0.039	87.388	0.528	0.599

p-value < 0.001 = ***, p-value < 0.01 = **, p-value < 0.05 = *, p-value > 0.05 = n.s.; ar: articulation rate, ed: Euclidean distance, fdisp: formant dispersion

For males, f0 range was a significant predictor of openness scores (Table 8). This result was found because male speakers who used a wider f0 range received higher openness scores. For female speakers, there were several acoustic variables that accounted for the scores of openness (Table 8), which contrasted with previous studies that have not usually found a clear marker of openness. Both PC2 and PC3 were significant predictors of openness scores, similar to the result of extraversion. The result of PC2 indicates that a voice quality associated with greater values of spectral tilt measures (i.e., breathier or more modal voice) and low f0 reduces the degree of openness perceived from the voice. It is difficult to understand the negative correlation between H2kHz*–H5kHz* and PC2, as explained above. PC3 also explained the openness scores; female speakers with more modal or breathier voice received higher openness scores, which is difficult to fully understand, as explained above. F0 range was also significant, suggesting that female speakers whose f0 range was wider received higher openness scores. Similar to the result of extraversion, formant dispersion was found to be a significant factor affecting openness scores of female speakers, with speakers with more closely located formant frequencies (i.e., longer vocal tract length) receiving higher openness scores. Overall, the results were highly similar for openness and extraversion ratings of female speakers.

**Table 8:** Results of the mixed-effects analysis for the ratings of openness.

		Estimate	Std. Error	df	t value	Pr(>|t|)
**Male**	(Intercept)	2.826	0.065	9.041	43.396	0
	PC1	-0.027	0.036	62.339	-0.756	0.452
	PC2	0.015	0.026	80.859	0.57	0.57
	PC3	-0.006	0.036	68.004	-0.156	0.876
	PC4	-0.023	0.028	69.548	-0.813	0.419
	**F0range**	0.066	0.026	77.507	2.507	0.014*
	ar	0.029	0.028	76.782	1.035	0.304
	ed	-0.011	0.025	63.046	-0.458	0.648
	fdisp	-0.013	0.034	79.533	-0.379	0.706
**Female**	(Intercept)	2.948	0.067	12.788	43.864	0
	PC1	0.001	0.033	93.912	0.037	0.97
	**PC2**	-0.096	0.04	92.348	-2.416	0.018*
	**PC3**	0.085	0.032	90.794	2.681	0.009**
	PC4	-0.034	0.033	95.926	-1.038	0.302
	**F0range**	0.057	0.027	88.487	2.152	0.034*
	ar	0.024	0.032	76.972	0.76	0.449
	ed	-0.011	0.028	87.022	-0.413	0.681
	**fdisp**	-0.089	0.031	90.018	-2.911	0.005**

p-value < 0.001 = ***, p-value < 0.01 = **, p-value < 0.05 = *, p-value > 0.05 = n.s.; ar: articulation rate, ed: Euclidean distance, fdisp: formant dispersion

## Discussion

The present study aimed to identify acoustic markers of personality that is perceived from Korean utterances produced in formal communicative contexts between unfamiliar individuals. The speakers in the present study were playing the role of a customer service employee in an experimental setting. Their speaking styles differed from one another; some sounded more professional or fluent, and some sounded more polite than others. We examined how these different speaking styles and characteristics are perceived in terms of the Big-Five personality traits. We investigated this using Korean in which speakers’ personality traits may be expressed and perceived differently from other cultures. Moreover, we examined not only global acoustic characteristics (e.g., f0 range, articulation rate), but also segmental properties (e.g., vowel expansion) and voice quality to cover a wider range of speech characteristics than previously examined. As a result, we found acoustic markers of personality that had not been found in previous research while replicating some of the previous results.

Firstly, we found that PC1 comprising of HNRs, CPP, and f0 affected the ratings of conscientiousness for female speakers; more modal and higher-pitched female voices increased the scores of conscientiousness. It suggests that female speakers who used a modal and higher-pitched voice (i.e., likely a less creaky voice) were perceived as being an individual who was more hard-working, capable, and responsible in the present study. Previous research has shown that creaky voice (“vocal fry”) is perceived negatively in the U.S. especially when used by young female speakers [58]; vocal fry in female voices was perceived as less competent, less educated, less attractive, less trustworthy, and less hirable. In Korea, the use of vocal fry in young women has not been reported. More research will thus be needed to understand how a creaky voice used by female speakers is perceived in Korea, but it is interesting to note that in the current study, less modal voice with lower f0 was similarly perceived as less conscientious in Korean women. Waaramaa et al. [30] also found that creaky voice was perceived as indicating negative personality traits such as laziness or a lack of enthusiasm by both native and non-native speakers of Finnish. However, in the present study, this result of voice quality was not found for male speakers. For them, a faster speaking rate increased the degree of perceived conscientiousness.

PC1 was also found to be significant for the perception of neuroticism in female speakers. The association with high pitch has already been reported in previous research [21, 23], but this finding also suggests that more modal voice (less noise in the source) increases the degree of neuroticism perceived from female voices. In addition, neuroticism was also associated with an increasing speaking rate for both male and female speakers; when they spoke faster, the speakers were rated as being more neurotic. These findings related to the judgment of neuroticism have not been reported in previous research. In contrast, agreeableness of female speakers increased with a slower speaking rate. Similarly, slower speaking rate was used in polite speech in Korean [13], as well as in English speech that was perceived as being more benevolent [59]. We expected that speakers may modify their speaking style when assisting customers in order to enhance the clarity of their speech. While slower speaking rates were perceived as more agreeable, differences in vowel expansion did not explain scores of agreeableness or any other personality traits in the current study.

Throughout this study, we found several acoustic correlates of perceived personality that differed for female and male speakers (e.g., voice quality variables, formant dispersion, articulation rate). Differences depending on speaker sex have already been reported in previous research, especially in relation to voice attractiveness [3], but the differences we found were more substantial than expected. This suggests that different acoustic cues can be used for judging male and female speakers’ personalities. The present study demonstrated that voice quality was one of the strong predictors of female speakers’ personality impressions, whereas it was not for male speakers. Previous research has shown that a breathy voice contributes to female vocal attractiveness [51], whereas the effect of voice quality on male attractiveness is less clear [60, 61]. Our study presents a new finding that more sonorous voice quality combined with high pitch increases the degree of conscientiousness and neuroticism perceived from female speakers in formal communicative contexts. As described above, the current study also found that female speakers who spoke more slowly were perceived as being more agreeable. These results may reflect social stereotypes of female customer service employees in South Korea; professional and kind female customer service employees have a modal “news anchor”-like voice and tend to speak slowly for the customers. More research is needed to understand why the link between neuroticism and voice quality was only found for female speakers and how this finding can be interpreted in this communicative context.

Formant dispersion was also one of the significant predictors of extraversion and openness for female speakers only; a female speaker whose speech projected a larger body size (i.e., closely located formants) received higher scores of extraversion and openness. Formant dispersion has been associated with the perception of dominance and voice attractiveness in previous research [3, 51], such that female speakers with more widely dispersed formants (i.e., a smaller body size) are perceived as more attractive and less dominant. The link we found between extraversion and vocal masculinity in female voices was unexpected and should be understood in terms of the Korean culture; gender stereotypes stemming from Confucian culture still exist in Korea. Confucianism views women as being more introverted, passive, and warm, whereas men are considered to be stern and extroverted [62]. It is thus plausible that when listening to female speakers, the subjects associated greater vocal masculinity manifested by lower formant dispersion with a greater degree of extraversion. It is less clear why reduced femininity in female voices similarly led to an increase in openness. In fact, some studies have shown that extraversion and openness are highly related; the excitement-seeking facet of extraversion is most related to openness, and the feelings facet of openness is most related to extraversion [63]. It is possible that the raters of the current study found it difficult to assess openness due to the content of the utterances. They may have thus associated openness with features of extraversion as they are easier to attribute to speakers in this context. It is also possible that in Korea, men are more open than women in certain aspects, creating an association between openness and masculinity; gender-related differences in openness have been found in Western cultures [64–66]. Further investigation will be needed to understand how openness is interpreted within the sociocultural contexts of Korea, especially in relation to how speaker gender plays a role in perceiving openness from voices.

It remains unclear why listeners based their personality judgements on formant dispersion cues only for female speakers; they could have rated male speakers with more widely located formants (i.e., smaller body size) to be less extrovert and less open. These gender-related differences may have been partially caused by other biases in listener judgments. People may associate specific voice characteristics or speaking styles with the social stereotypes linked to the occupation, often dominated by women; more than 75% of call center agents are females in South Korea [67]. As a result, listeners may have relied more readily on those speech cues when judging the personality of female speakers.

In addition to formant dispersion, the present study also showed that f0 range was a significant predictor of both extraversion and openness. Specifically, a wider f0 range was associated with higher scores of extraversion and openness for both female and male speakers. An utterance’s f0 range is mainly associated with its intonation patterns. Specifically, linguistic information such as sentence type (e.g., statement vs. question) or focus can be expressed by different intonational structures and different types of tones in an utterance (see [68] for a review). In Korean, Intonational Phrase (IP) boundary tones (e.g., HL%, LH%) are used to convey different attitudes and emotions (e.g., polite, apologetic, annoyed; [69, 70]), as well as linguistic meanings (e.g., statement vs. question). A larger pitch movement can also signal paralinguistic meanings, such as emotional arousal [71, 72]; Jeon [73] reported that pitch expansion increased the perceived degree of valance and arousal in Korean IP boundary tones (e.g., HL%, LH%). It thus appears that speakers who used IP boundary tones with an expanded pitch range were perceived as individuals who express their feelings more actively in the current study, resulting in higher ratings of extraversion. In a future study, it will be interesting to investigate this association further by analyzing specific IP boundary tones used. A greater pitch range was likewise associated with higher ratings of openness in the current study. The use of formant dispersion cues was also uniformly found for both extraversion and openness ratings for female speakers. As explained above, these results were unexpected because previous research has shown that openness is difficult to attribute to a novel speaker and has found no clear acoustic markers of openness [20, 23]. Further studies are needed to understand the relationship between openness and extraversion ratings in the sociocultural contexts of South Korea.

The present study demonstrated that Koreans perceive the personalities of male and female speakers differently based on their speech, reflecting gender-related stereotypes in South Korea. It is also possible that the occupation-related bias (i.e., a higher representation of females in the occupation) in South Korea caused the gender-related perception differences. It will be interesting to conduct a similar investigation in different languages to explore cross-cultural and cross-linguistic differences in personality judgements. The findings of the current study will also be directly relevant to marketing strategies and the development of synthetic voices for VA applications. That is, the acoustic parameters of personality traits can be adjusted as needed to create a “persona” that suits the target service (e.g., AI robots for older populations, AI bankers), or to develop a synthetic voice that is preferred for customer service applications (e.g., one that sounds more agreeable and conscientious). Understanding the acoustic manifestations of different personality traits could also help develop an automatic evaluation system for speech synthesis, similar to the method used in Wagner et al. [74]. Such a system will overcome the limitations and costs associated with having human listeners rate the overall quality of synthetic speech subjectively (i.e., the Mean Opinion Score).
